# Green Extraction of Natural Products: Concept and Principles

**DOI:** 10.3390/ijms13078615

**Published:** 2012-07-11

**Authors:** Farid Chemat, Maryline Abert Vian, Giancarlo Cravotto

**Affiliations:** 1 Université Avignon et des Pays de Vaucluse, INRA, UMR408, Avignon 84000, France; E-Mail: maryline.vian@univ-avignon.fr; 2 Dipartimento di Scienza e Tecnologia del Farmaco, Università di Torino, via P. Giuria 9, Torino 10125, Italy; E-Mail: giancarlo.cravotto@unito.it

**Keywords:** green extraction, alternative solvents, innovative process, natural products

## Abstract

The design of green and sustainable extraction methods of natural products is currently a hot research topic in the multidisciplinary area of applied chemistry, biology and technology. Herein we aimed to introduce the six principles of green-extraction, describing a multifaceted strategy to apply this concept at research and industrial level. The mainstay of this working protocol are new and innovative technologies, process intensification, agro-solvents and energy saving. The concept, principles and examples of green extraction here discussed, offer an updated glimpse of the huge technological effort that is being made and the diverse applications that are being developed.

## 1. Introduction

The extraction of natural products, for example in the perfume industry, was considered “clean” when compared with heavy chemical industries, but researchers and professional specialists found that its environmental impact is far greater than first appeared. The overall environmental impact of an industrial extraction cycle is not easily to estimate; however it is known that it requires at least the 50% of the energy of the whole industrial process. In spite of the high energy consumption and the large amount of solvents, often the yield is indicated in decimals. For example, a single milliliter of rose absolute that weighs less than 1 gram requires not only 1 kg of fresh roses as raw material but also a large quantity of solvents (*n*-hexane, alcohol), energy (fossil) and water as cooling agent.

Extraction of natural products has been used probably since the discovery of fire. Egyptians and Phoenicians, Jews and Arabs, Indians and Chinese, Greeks and Romans, and even Mayas and Aztecs, all possessed innovative extraction processes (maceration, alembic distillation, *etc.*) used even for perfume, medicine or food. Nowadays, we cannot find a production process in the perfume, cosmetic, pharmaceutical, food, bio fuel, or fine chemicals industries, which does not use extraction processes, such as (maceration, steam or hydro-distillation, pressing, decoction, infusion, percolation and Soxhlet extraction). In the food industry, besides the well established huge extraction processes of sugar beet and sugar cane, and the preparation of decaffeinated tea and coffee, many formulations have been developed by adding plant extracts and nutraceuticals concentrates. Bioactive compounds or their precursors (antibiotics, chemo-preventive agents, alkaloids, *etc*.) are extracted by the pharmaceutical industry, either with conventional methods or modern technologies. Recent trends in extraction techniques have largely focused on finding solutions that minimize the use of solvents. This, of course, must be achieved while also enabling process intensification and a cost-effective production of high quality extracts.

The challenges launched by the competitiveness of the globalized market and environment protection strongly require technological innovations that break away from the past rather than simple continuity. The 12 principles of green chemistry were quickly matched by the 12 principles of green engineering that form the basis of modern “sustainable” processes [1]. The European scenario is being dramatically changed by the directive REACH (Registration, Evaluation, Authorisation and Restriction of Chemicals) concerning chemical substances, either as such, or entering into products or manufactured objects, while IPPC (Integrated Pollution Prevention Control) is aimed at reducing the contribution of industry to non-sustainable development. This directive puts the spotlight on processes by defining the notion of BAT (Best Available Technology) for each professional sector. It puts product and process together definitively, according to the adage “You won’t make tomorrow’s products using yesterday’s processes”. In this context, the development of green technologies and the use of renewable raw material occupy a central role in environmental-friendly processes. An R & D policy to be applied even to traditional solid-liquid extraction (SLE) processes would be advantageous.

## 2. Definition of Green Extraction

A general definition of green chemistry is the invention, design and application of chemical products and processes to reduce or to eliminate the use and generation of hazardous substances. In relation to green extraction of natural products, this definition can be modified as follows: “*Green Extraction is based on the discovery and design of extraction processes which will reduce energy consumption, allows use of alternative solvents and renewable natural products, and ensure a safe and high quality extract/product*”.

Three major solutions have been identified to design and demonstrate green extraction on laboratory and industrial scale to approach an optimal consumption of raw materials, solvents and energy: (1) improving and optimisation of existing processes; (2) using non-dedicated equipment; and (3) innovation in processes and procedures but also in discovering alternative solvents.

## 3. The Six Principles of Green Extraction

The listing of the “six principles of Green Extraction of Natural Products” should be viewed for industry and scientists as a direction to establish an innovative and green label, charter and standard, and as a reflection to innovate not only in process but in all aspects of solid-liquid extraction:

**Principle 1:** Innovation by selection of varieties and use of renewable plant resources.
**Principle 2:** Use of alternative solvents and principally water or agro-solvents.
**Principle 3:** Reduce energy consumption by energy recovery and using innovative technologies.
**Principle 4:** Production of co-products instead of waste to include the bio-and agro-refining industry.
**Principle 5:** Reduce unit operations and favour safe, robust and controlled processes.
**Principle 6:** Aim for a non denatured and biodegradable extract without contaminants.

The principles have been identified and described not as rules but more as innovative examples to follow, discovered by scientists and successfully applied by industry.

### 3.1. Principle 1: Innovation by the Selection of Varieties and the Use of Renewable Plant Resources

The increasing demand of natural products and extracts is leading to the over-exploitation of natural plant resources. History reports several examples of plant extinction because of overutilization; the preservation of biodiversity is therefore mandatory in the respect of future generations. In green extraction, fully renewable resources have to be favoured either with intensive cultivation or *in vitro* growth of plant cells or organisms.

A quarter of current medicines are extracted from plants, the anti-cancer paclitaxel (Taxol^®^) extracted from the bark of the western yew (*Taxus brevifolia*) is the best known example. During the 1970s no less than 30 tonnes of bark were collected for clinical trials: 10 kg of dry bark produce only 1 g of taxol after extraction and purification.

**Figure 1:**
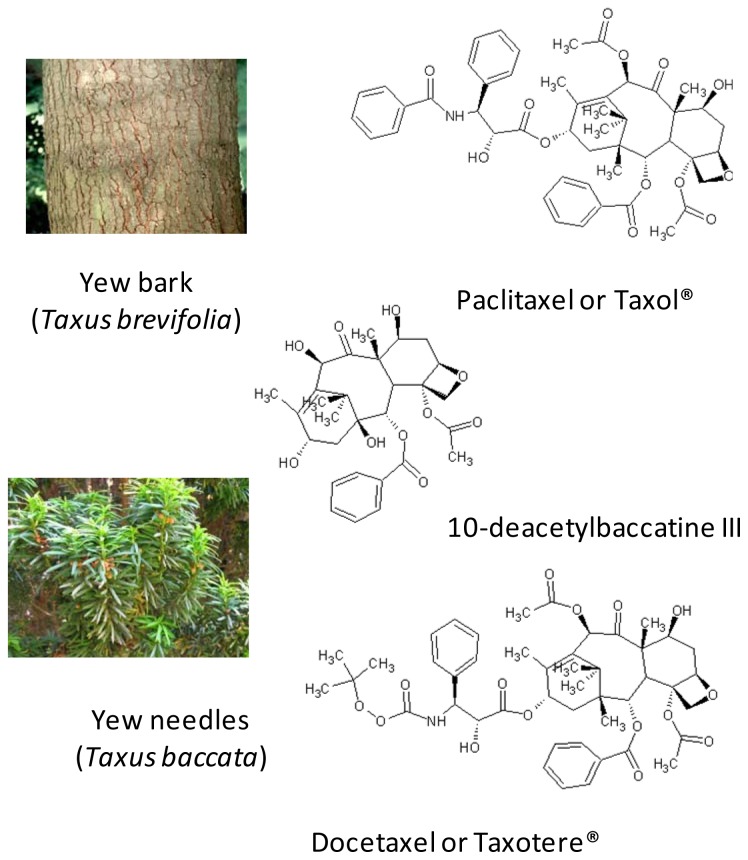
Green Extraction of Docetaxel.

A large number of research projects have therefore been aimed at finding alternatives to felling trees of this threatened species. Since 1980 paclitaxel and docetaxol (Taxotere^®^) are prepared by semisynthesis from the natural precursor, 10-deacetylbaccatine III, extracted from needles and branches (renewable resource) of different yew tree species. Figure 1 presents the green route to docetaxol instead of the conventional procedure which uses non-renewable plant resources.

The large-scale uncontrolled harvesting of natural resources thus carries the risk of making the species rare or even extinct. Harpagophytum or “Devil’s claw” is a case in point; this is greatly sought-after by pharmaceutical laboratories to treat rheumatism. This plant is particularly under threat: it only grows in the Kalahari Desert (Namibia) and, with 600 tonnes exported every year, extinction is more than likely.

**Figure 2:**
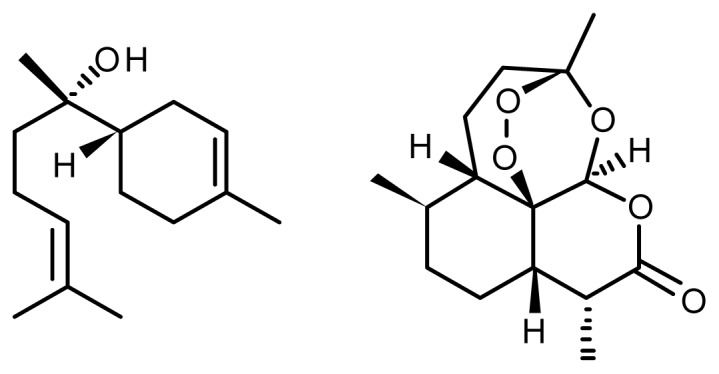
(−)-alpha-Bisabolol (**left**) and artemisinin (**right**).

The natural occurrence of (−)-alpha-bisabolol (Figure 2) is mainly from candeia plant (*Eremanthus erythropappus* (DC) MacLeish) which grows in the Atlantic Brazilian rainforest, in the south of Minas Gerias State, a plant in precarious ecological situation. The sustainable supply of candeia oil is seriously under threat. Symrise, Revlon and Rossman are amongst the 187 companies to have launched cosmetic products containing alpha-bisabolol in the last two decades, a sesquiterpene with marked anti-inflammatory, antibacterial and antifungal properties. In a move to integrate sustainability into its corporate social responsibility strategy, Symrise recently decided to stop harvesting natural bisabolol from the candeia tree. Other species, such as sandalwood and rosewood, are also over-exploited and could become extinct. When adopting a policy of green extraction, the relevant choice is to use only cultivated plants and not plants taken from their natural habitat; only controlled crops can contribute to conserving biodiversity. A big effort is running in the natural selection of varieties with much higher concentrations of active ingredients.

A topical example is the production of artemisinin, an anti-malarial substance isolated from annual wormwood, *Artemisia annua* L., originating from Asia. The active principle of this plant, a sesquiterpene lactone with a peroxide bridge, is present in the aerial parts of the plants at a concentration in the order of 0.01 to 0.05% (Figure 1). Extracting this active substance turns out to be hardly cost effective because of the low concentrations in the plant. Much experimental work has been done to produce varieties of *Artemisia annua* L. with concentrations of artemisinin greater than 1%.

**Figure 3:**
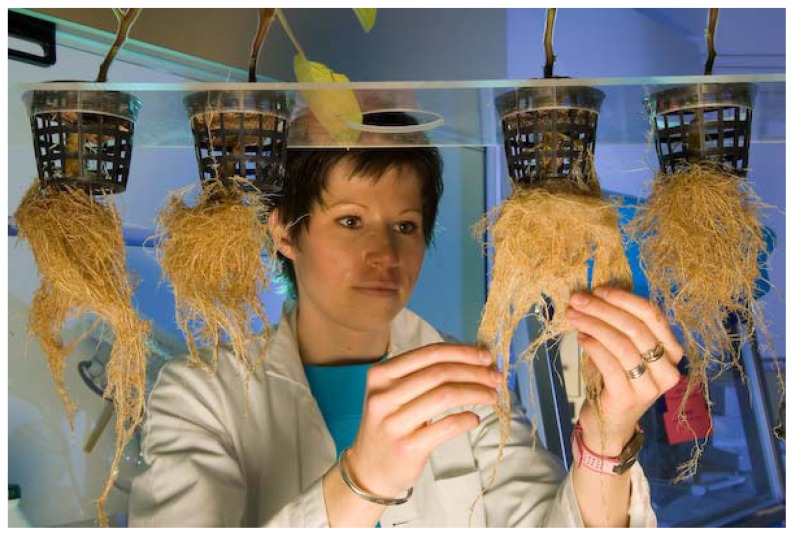
Plant milking technology. (PAT plant milking^©^. Photograph: Philippe Psaïla).

A new technology “plant milking” has been developed for the production and extraction of substances of interest without destroying the plant. Plants are grown in a greenhouse in a liquid medium and the secretion and exuding of the substances through the roots in the culture medium are triggered by physical, chemical or biological stimulation. The substances are then collected by standard extraction and purification methods. This process is more directed to the production of active principles from rare plants, whose chemical synthesis is difficult and costly (Figure 3). “Plant milking” is thus a route to production that respects biodiversity. This process has enabled the production of, among others, tropane alkaloids of pharmaceutical interest from *Datura innoxia*. In this case, harvesting yields of three times more secondary metabolites in one year than extraction from field-grown plants with an equal surface area have been obtained.

Good results have also been obtained with garden rue (*Ruta graveolens*), which contains furocoumarins, substances used to treat eczema and psoriasis, and with edelweiss, rich in antioxidants. In the case of the yew, this novel harvesting method, “plant milking”, yields much larger quantities of paclitaxel than traditional harvesting methods. If we could manage to use this technique on a large scale, a few hundred greenhouses would be sufficient to satisfy world demand for paclitaxel for one year [2].

### 3.2. Principle 2: Use of Alternative Solvents and Principally Water or Agro-Solvents Issued from Agricultural Resources

Current regulations have a progressive direct impact in diminishing the consumption of petrochemical solvents and Volatile Organic Compounds (VOCs). Manufacturers that use organic solvents have to show the absence of risk during extraction and to demonstrate the safety of ingredients as regards to solvent traces. Most organic solvents are flammable, volatile, and often toxic and are responsible for environmental pollution and the greenhouse effect. Safety, environmental and economical aspects are forcing industry to turn to greener solvents.

Among green solvents, the agro-or bio-solvents play an important role for the replacement of petrochemical solvents. They are a renewable resource produced from biomasses such as wood, starch, vegetable oils or fruits. These bio-solvents have a high solvent power, are biodegradable, non-toxic and non-flammable. Their limitations and drawbacks are due to cost, high viscosity, high boiling point and generation of off-flavours.

Ethanol is the most common bio-solvent, obtained by the fermentation of sugar-rich materials such as sugar beet and cereals. Although it is flammable and potentially explosive, ethanol is used on a large scale because it is easily available in high purity, it has a low price and it is completely biodegradable. In this category of agro- or bio-solvents, we can also find terpenes extracted from pine (α-pinene) or citrus fruit (*d*-limonene). Because of their low polarity and their very high solvent power, *d*-limonene can be used for the extraction of fat and oils [3].

Methyl esters of fatty acids of vegetable oil (soya, cocoa and rapeseed) can also replace petrochemical solvents. Besides being biodegradable and non-toxic, methyl esters of fatty acids do not emit VOCs (volatile organic compounds), they have technical performances comparable with those of petrochemical solvents.

Glycerol, a by-product from the trans-esterification of vegetable oils, is very common in the cosmetic industry as a solvent for maceration of herbs and spices. Ionic liquids are already applied in green extraction of natural products for their solvent power, high chemical and thermal stability, and as a non-flammable and non-VOC solvent. For example, in the case of extraction of artemisinin, by using ionic liquids, pure compound is obtained after simple precipitation [4].

The polar nature of water also makes it possible for use as an extraction solvent in the field of natural water-soluble products such as proteins, sugars and organic acids, as well as for inorganic substances. Pressurised hot water extraction (PHWE) or sub-critical water is one of the most promising modern green extraction techniques and methods especially in a dynamic mode. Its outstanding feature is the easy manipulation of the dielectric constant (ɛ_r_) of water, that can be made to vary over a wide range just by changing the temperature and pressure. Thus, at ambient temperature and pressure, water has a dielectric constant of *ca.* 80, which makes it an extremely polar solvent. This value can be drastically lowered by raising the temperature under moderate pressure. For instance, subcritical water at 250 °C and a pressure just over 4 MPa has a ɛ_r_ value of 27, which is close to ethanol, and is suitable for extraction of low-polarity compounds [5].

A recent green innovation in water extraction entails the use of cyclodextrins in solution for their ability to form inclusion complexes between bioactive compounds and their peculiar hydrophobic cavity. The ultrasound-assisted extraction of resveratrol and other polyphenols from the milled roots of *Polygonum cuspidatum* has been efficiently carried out in a water solution of β-cyclodextrin (1.5%). The selective inclusion properties of cyclodextrins toward phenolic stilbenes gave a much cleaner analytical extract profile if compared with that obtained with methanol. Thanks to polyphenol encapsulation, this extract showed excellent water dispersibility, higher stability and antioxidant power [6].

Extraction with supercritical CO_2_ has been developed since the 1970s and has now reached a mature stage. Using this technique, pure perfumes, fragrances and active ingredients can be obtained with no traces of solvent. CO_2_ is a non-flammable odourless gas produced during the burning of fossil fuels, by alcoholic fermentation and also through human and animal respiration. The technique of extraction with supercritical CO_2_ uses compressed gas at a pressure of up to 300 MPa at a moderate temperature (30–40 °C), to replace organic solvents such as hexane. Supercritical CO_2_ extraction is being applied in several sectors such as food, cosmetics and pharmaceutical industry. It is used for weakly polar compounds of low molecular weight such as carotenoids, triglycerides, fatty acids, aromas, *etc*. The main drawbacks remain its high initial investment and difficulties to perform continuous extractions [7].

A new microwave solvent-free extraction technique called Microwave Hydrodiffusion and Gravity (MHG) has been used for extraction of essential oils, colours and antioxidants. It is an original “upside down” microwave alembic combining microwave heating and earth gravity at atmospheric pressure. Based on a relatively simple principle, this method also involves placing the plant material in a microwave reactor, without adding any solvent or water. The internal heating of the *in situ* water within the plant material distends the plant cells and leads to the rupture of glands and oleiferous receptacles. The heating action of microwaves thus frees essential oil and *in situ* water which are transferred from the inside to the outside of the plant material, and drop by gravity out of the microwave reactor [8]. Table 1 summarizes alternative solvents for Green extraction.

### 3.3. Principle 3: Reduce Energy Consumption by Energy Recovery and Using Innovative Technologies

Extraction is particularly affected by environmental and economical factors that require a massive reduction of energy consumption and wastes produced. There are four routes to minimise energy consumption: optimising existing processes, recovery the energy liberated during the extraction process, assisting existing processes with intensification, and a full process innovation.

Hydrodistillation is an ancient technique for extraction of essential oils. It is used worldwide for its simplicity but requires high energy consumption for heating and cooling. When the process is carried out under moderate pressure, distillation time is reduced by a factor of 2 or 3, with a reduced consumption of steam and hence reduced energy consumption. Some aromatic plants such as sandalwood, cloves, or the rhizomes of vetiver, ginger and iris, need more than 24 hours with atmospheric distillation but less than 3 hours with pressure steam-distillation at 0.5 MPa. In general, prolonged heating at high temperature may reduce the quality of the essential oil by the formation of undesired side products.

A reduction of energy consumption can be achieved by recuperating the heat liberated during vapour condensation. A prototype of eco-evaporator able to recover up to 55% of the energy was developed in the south of France for steam-distillation of lavender. The overall saving in thermal energy was around 80% with no impact on the product’s quality.

Ultrasound assisted hydrodistillation can contribute to intensify and to improve the efficiency of essential oil extraction and considerably reduce extraction time and energy used [9]. Ultrasound assisted hydrodistillation has been used for extracting essential oil of yuzu, a Japanese citrus fruit. With this process extraction yield is 44% higher than the conventional method.

Once the extraction process has become a limiting step for the industrial development, the innovation is mandatory, looking for new solvents, new activation techniques or new technologies. Several emerging techniques applied to extraction are helping to reduce the number of stages, the extraction time and thus the energy consumption. An example is the use of pulsed electric fields, activation by microwaves or ultrasound. In setting up extraction operations in complex continuous installations, the limitations imposed by the type of process can be overcome and new products can even be offered. A case in point is the twin-screw extruder used for fractionating vegetable material, which handles take-up, thermal treatment, extraction under mechanical pressure and separation. There are also modified pulsed columns to treat the solid matter and which offer new operational ranges [10].

### 3.4. Principle 4: Production of Co-Products Instead of Waste to Include the Bio and Agro Refining Industry

Besides the extraction products such as the ingredients or the solvent recovery, a wide range of other materials are generated during industrial processes: by-products, co-products, or waste:

- Waste is any material that industry has the only choice to eliminate by waste disposal centre, incineration, or landfill.
- By-product is a residual product that appears during the extraction process. It is unintentional and unpredictable. It can be used directly or be an ingredient in another production process to manufacture another finished product. By-products have economic value, for example, oil cakes (rape, sunflower, flax), cereal grain (wheat, barley), beet pulp, potato fibre and proteins, *etc*.
- Co-product is a material, intentional and inevitable, created during a single manufacturing process and at the same time as the main product. The main final product and co-product must always meet specifications for their characteristics, and each may be used directly for a particular application.

The “Bio-refinery” concept is becoming widely accepted as the world’s natural resources are being used up and can be considered as a facility that combines the biomass conversion process with equipment to produce a wide range of bio-based products such as biofuels and biomaterials. However, it is still a largely unexplored territory and presents many research opportunities for the production of high value-added compounds from agricultural and forest residues.

This is a new green strategy in the transformation of agro-products that yields a range of target products from a given biomass, unlike the traditional approach which is generally designed to give a single product. With this concept the entire plant material is used in an integrated approach. Plants are made up of an enormous number of substances that may be refined: each constituent of the plant can be extracted and functionalised to produce green fuels, building materials, packaging, maintenance products, beauty creams, *etc*.

There are many examples of bio-raffinery valorisation using extraction of by and co-products. For example, rosemary is an aromatic plant well known for its antioxidant properties due to polyphenolic compounds such as carnosol and rosmarinic and carnosic acids. After distillation of the essential oil, the residue can be re-processed to extract natural antioxidants, in very high demand by the agro food and cosmetics industries. The polyphenolic extract of rosemary can be used in the meat industry to inhibit oxidation of lipids responsible for modifying the aspects (aroma, colour) that affect shelf life of the product.

The transformation of oil seeds into food oil generates a large volume of by-products. Oil cakes, rich in proteins, from pressing seeds, are generally used for animal feed. The crude oil (generally about 3–10% in oil cakes) can be transformed into methyl esters by trans-esterification to produce biodiesel.

### 3.5. Principle 5: Reduce Unit Operations through Technical Innovation and Favour Safe, Robust and Controlled Processes

To be competitive industries involved in extraction of natural products (perfume, cosmetic, pharmaceutical, food, and bio-fuel) have to combine process intensification with cleaner and safer extraction protocols. Process intensification covers all developments of the new equipment, techniques, or procedures that bring significant progress in comparison with the current production methods. The challenges of the industrial development of intensified processes are multiple: more compact production units and a reduced number of unit operations, energy and raw material savings, process safety control, reduction in waste and ecological footprint.

Solvent extraction of natural products is not limited to a single unit operation; it is really a chain of processes. For example, to extract β-carotene from carrots, the procedure is as follows: (1) dry the vegetable matter (apolar solvent such as n-hexane could penetrate the matix); (2) grind or shred the vegetable matrix (to increase the contact area); (3) extract by contacting solvent and matrix; (4) separate liquid and solid phases; and (5) evaporate the liquid phase containing β-carotene and recycle the solvent. And finally, it is often necessary to carry out molecular distillation or vacuum drying to eliminate residual traces of undesirable solvents according to the regulations in force.

Reducing the number of steps in a process chain leads to a reduction in costs and better use of energy. A single-stage process would appear to be ideal. The process of extraction by supercritical fluids has the advantage of using a clean solvent and also of obtaining an extract by a technology having a minimum number of individual operations. After extraction, releasing the pressure, CO_2_ is eliminated and recycled, and isolated extract is free of all traces of solvent. Extraction by supercritical fluids has always suffered from being seen as a more expensive technology than traditional ones. However, if the whole chain of processes is taken into account, and not the individual operation itself, we see that this technology requires few individual operations. Moreover, it does not involve the use of toxic solvents and represents a reduction in the energy used from the extraction step to the distillation/recycling of the solvent.

An original innovation, in the green extraction of natural products, will be the transportability of the extraction process. Usually, industries where the raw material is transformed are often very far from where the plant species are grown and harvested. The carbon cost of transporting this raw material to the extraction site is by no means negligible. The “green” distillation of lavender is an example of a “transportable” process. As it is gathered, the plant is immediately chopped up in a mobile kettle which is mounted directly on a boiler. The fresh lavender is distilled on the spot, avoiding transport to the distillery and drying of the lavender. However, this process does yield a characteristic olfactory product with green aromas. In the “green” distillation process, all steps from cutting to distillation are simplified, with a consequent reduction in handling while respecting essential qualities of the plant [11].

When evaluating the potential environmental impact of an extraction process, the use of resources has to be quantified together with the environmental emissions associated with the system. In such systems, the transport of raw material is recognised as playing a predominant role; it consumes the highest amount of resources and causes the most pollution. Consequently, the development of mobile transportable “green extractor” is a way of considerably reducing the ecological footprint of the extract/product. A teaching example is the extraction of essential oil from olibanum, more commonly known as frankincense. This essential oil is obtained from a resin from the bark of a shrub originally from the area surrounding the Red Sea, in Somalia and Arabia. To collect the resin, fine incisions are made in the bark, and drops of sap appear and dry in large, odorous yellow droplets. The resin is sent to Grasse (France) where it is processed and transformed into essential oil. This resin could be processed on the spot with mobile equipment rather than sending it abroad (thousands of kilometres).

We may allude to the novel by Paul Féval and say: “Si tu ne viens pas à Grasse, Grasse ira à toi!” (If you don’t come to Grasse, Grasse will come to you!).

### 3.6. Principle 6: Aim for a Non Denatured and Biodegradable Extract without Contaminants with “Green” Values

The whole extract (phytocomplex in phyotherapy), is a mixture of bio-active compounds and other substances that can affect solubility and absorption. The composition of the extract can therefore influence the flavour and bioavailability of alkaloids, polyphenols, flavonoids, terpenes and meroterpenoids, by the presence of natural carriers such as saponins, phospholipids, *etc*. Nevertheless the constraints introduced by the regulations often require different preparation rules. Regulations on essentials oils in cosmetics are relatively succinct, whereas in the pharmacy and food industries they show more drastic constraints. Pharmaceutical specialities based on essential oils must meet the definition of plant-based medicine. They must therefore comply with the regulations governing these medicines, and be registered as a traditional plant-based medicine. In the food sector, essential oils are most often used as food flavourings and fall under the regulations of European directives.

**Figure 4:**
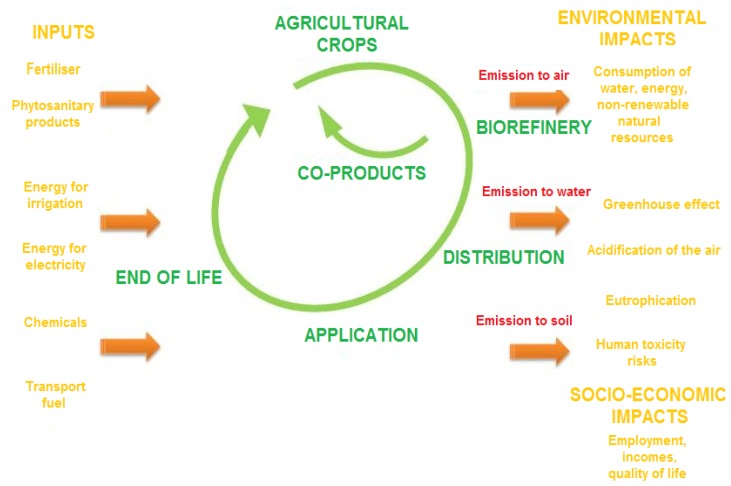
Life Cycle Analysis of extraction of naturals products.

To meet the requirements of the market and of the regulations, the extract must meet a number of quality criteria; contrary to some popular misconceptions, the “natural” state of the extract is no guarantee of its harmlessness to man and the environment. In particular, the extracts must be obtained from precisely identified raw materials, inspected according to defined procedures to guarantee their naturalness. They must also have precise physico-chemical properties and be properly stored. The geographical origin and the environmental conditions under which the raw material was obtained (whether non authorised pesticides were used, for example) are also parameters to be taken into account. And lastly, the extract should be free of all pollutants such as pesticide residues, heavy metals, mycotoxins, *etc*. Life cycle analysis (LCA) is based on the concept of sustainable development, providing an effective and systematic means of assessing the environmental impacts of a product, a service or a process (Figure 4). This assessment tool is applied to various categories of environmental impact. It enables the quantification of potential impacts on the environment of a system that includes all of the activities associated with a product or a service, from the extraction of the raw materials up to the end of life processing (waste disposal centre, incineration, recycling, *etc*.), including the distribution and use. This method consists of listing the consumptions of natural resources, energy and environmental emissions (water, air and soil). The flows of matter entering and leaving at each step of the life cycle are inventoried (life cycle inventory, LCI). This inventory of base flows shows the quantities of polluting substances emitted and the resources extracted during the life cycle. From the results gathered, the environmental impacts are assessed by calculating the contribution of each of the flows to the various environmental impacts studied. The most common impacts taken in these studies are the greenhouse effect, eutrophication, acidification, ozone layer destruction, ecotoxicity, photochemical pollution, human health, the exhausting of natural resources, *etc*. The results of an LCA are expressed in the form of a set of figures showing both the potential impacts (of the type “X kg of CO_2_ equivalents for the greenhouse effect”, “Y kg of H^+^ equivalents for acidification” and so on) and physical flows (“Z MJ of non-renewable energy”, “W kg of ordinary waste”, *etc*.). To quantify the effect of “green extract” on sustainability, it is better to look at the life cycle analysis, not of the product alone, or of the isolated extraction process as an individual unit operation, but of the whole supply chain, including the production of the plant and its harvesting and the recycling of the by-products and biodegradability of the products obtained [12].

## 4. Conclusions

Extraction, according to the six principles of green extraction of natural products, is a new concept to meet the challenges of the 21st century, to protect both the environment and consumers, and in the meantime enhance competition of industries to be more ecologic, economic and innovative. Within this green extraction approach, the concept of the green extract is introduced—an extract obtained in such a way to have the lowest possible impact on the environment (less energy and solvent consumption, *etc*.), and whose eventual recycling would have been planned for (co-products, biodegradability, *etc*.). This green extract should be the result of a whole chain of values in both senses of the term: economic and responsible, starting from the production and harvesting of the plant, the transformation processes of extraction and separation together with formulation and marketing. This “green extract” could be identifiable in the future by a European or International Label or Standard.

## Figures and Tables

**Table 1:** Alternative solvents for Green Extraction.

Solvent	Extraction Technique (Application)	Solvent Power			Health & Safety	Cost	Environmental Impact

		Polar	Weakly Polar	Non-Polar			
**Solvent-free**	Microwave Hydrodiffusion and Gravity (antioxidants, essential oils)	+++	+		+++	+	+++
	Pulse Electric Field (antioxidants, pigments)	+++	+		+++	+	+++

**Water**	Steam distillation (essential oils)	++	+		+	++	+
	Microwave-assisted distillation (essential oils)	+++	+++	+	+	+	++
	Extraction by sub-critical water (Aromas)	+	++		+	+	+

**CO_2_**	Supercritical fluid extraction (decaffeination of tea and coffee)	−	+	+++	+	+	+

**Ionic liquids**	Ammonium salts (Artemisinin)	−	+	+++	−	−	++

**Agrosolvents**	Ethanol (pigments and antioxidants)	+	+	−	−	++	+
	Glycerol (polyphenols)	+	+	−	−	+	+
	Terpenes such as *d*-limonene (fats and oils)	−	−	++	−	+	+

**Petrochemical solvents**	*n-*Hexane (fats and oils)	−	+	+++	−−−	++	−−−
